# The Three-Dimensional Morphological Effects of Captivity

**DOI:** 10.1371/journal.pone.0113437

**Published:** 2014-11-19

**Authors:** Adam Hartstone-Rose, Hannah Selvey, Joseph R. Villari, Madeline Atwell, Tammy Schmidt

**Affiliations:** 1 Department of Cell Biology and Anatomy, University of South Carolina School of Medicine, Columbia, South Carolina, United States of America; 2 Department of Anthropology, University of South Carolina, Columbia, South Carolina, United States of America; 3 Department of Environmental Science and Policy, George Mason University, Fairfax, Virginia, United States of America; 4 Mammals, Zoo Atlanta, Atlanta, GA, United States of America; Institut Pluridisciplinaire Hubert Curien, France

## Abstract

Many captive animals are fed diets that are drastically different in mechanical properties than their wild diet. Most captive pantherines are fed a nutritionally supplemented diet consisting almost entirely of ground meat. While many zoos supplement this diet with bones, the fact remains that large captive felids are fed diets that require substantially less masticatory effort than those of their wild counterparts. The osteological effects of this dietary difference have not been fully evaluated. To this end, we compared linear measurements and 3D geometric morphometric landmarks of captive and wild lions and tigers. Using Principal Component (PC) analysis of the linear measurements, not only were the sexes and species statistically distinct, but so too was the population clearly divisible in terms of captivity status. The 3D analysis supported these findings: although the most influential variable in the sample (PC1, 21.5% of the variation) separates the two species, the second most influential contributor (PC2) to the overall skull shape is driven not by the sex differences in these highly dimorphic species, but rather by their captivity status. In fact, captivity status drives nearly twice as much of the 3D variation as sexual dimorphism (14.8% vs. 8.0% for PC2 vs. PC3). Thus the shape is influenced nearly twice as much by whether the animal was captive or wild than by whether it was male or female. If a causal relationship can be demonstrated between dietary mechanical properties and morphology, people who oversee the diets of captive carnivores should consider modifying these diets to account for not only nutritional but also the mechanical properties of a carcass-based diet as well. In addition to the husbandry implications, our analyses show the ways in which captive specimens are different than their wild counterparts – findings that have implications for morphologists when considering anatomical samples.

## Introduction

Comparative morphologists tend to exclude captive animals from their research because of perceived distortions in these animals’ anatomy. Although morphological differences between captive and wild animals have been observed for a very long time (e.g., [1], [2]) including in some of the oldest captive specimens on record [3], these distortions have never been quantified in terms of their three dimensional shape, nor have the reasons for these observed morphological abnormalities been fully explored. If some aspect of captive husbandry that is negatively affecting the well-being of the animals can be identified, it may be possible to modify “best practices” to allow animals to live more naturalistic lives and exhibit morphology that is more similar to that of their wild counterparts. Additionally, quantifying the morphological effects of captivity will help morphologists make decisions about specimen selection and address sources of sample based bias.

### The Standard Zoo Diet

Most captive facilities provide felids with a diet of ground meat supplemented with vitamins. Many companies advertise commercial meat products that contain muscle with vitamin and mineral supplements, or supplements that, when added to meat products, will provide captive felids with the nutrition they need (e.g., [4]–[7]). Though commercial diets are based on the chemical components of whole prey, their lack of structural elements is often advertised as a selling point. For instance, one recent advertisement in the journal of the leading North American zoological organization, the Association of Zoos and Aquariums (AZA), prominently included in their “standards” that their diet includes “no bones, cartilage, organs, skin or connective tissues” ([5], p. 21). Given that wild lions and tigers predominantly consume vertebrate flesh off the bone including all associated connective tissues [8], [9], although these captive diets are nutritionally complete, they are structurally unnatural.

In recognition of the mechanical deficiency of these soft diets, bones are often presented separately as enrichment (e.g., [4], [10]). Some zoos, predominantly in Europe (for example, recently highly publicized at the Copenhagen Zoo in Denmark) practice carcass feeding, in which captive carnivores are fed freshly euthanized prey animals [11], [12]. The predators benefit from the carcass feeding because it yields a diet consistent with what hunting would yield in the wild: a diet that is nutritionally and mechanically complete ([12]). However, many other European and North American zoos have an aversion to this practice, considering the safety of the foods (e.g., predators may choke on carcass elements, the diet may spoil before it is fully consumed, or animals may fight over large articulated foods), and the reaction of the public [12]. The Copenhagen Zoo’s recent argument for the benefit of allowing their big cats to consume the meat of a surplus giraffe (*Giraffa camelopardalis reticulata*) sparked a global ethical controversy surrounding the euthanasia of a healthy animal that was then fed to its lions [11]. With the ethical consequences of feeding whole carcasses (i.e., food that looks more like other zoo animals than like hamburger meat), as well as the ease and established costs of the current system, most North American collections continue to feed their captive felines the heavily processed diet.

While some zoos have adopted enrichment for their felids, supplementing their diets weekly with previously-frozen rabbits, fish, mice, and some additional bones, these are still generally merely an optional dietary component [13]–[15].

### Psychological Effects of the Processed Diet

Though captivity does not provide animals the opportunity to forage or hunt as they would in the wild, the motivation to do so remains. This has been addressed in some zoos with feeder mechanisms that stimulate movement, such as lure courses for cheetahs (*Acinonyx jubatus*) and bungee cord feeders for lions and tigers. Not having the stimulation inherent in the act of hunting has been shown to ultimately lead to stereotypical behavior and abnormal behaviors such as over-grooming, lethargy, and pacing [16]. In an examination of the effects of various types of enrichment for captive felids, Skibiel and colleagues [17] tested the temperaments and overall health of six different species of captive felids by supplementing their typical (mechanically and psychologically un-stimulating) diets with bones, spices and frozen fish. This implementation caused a significant decrease in stereotypical behavior, suggesting that such enrichment encourages natural behavior that “may prevent physiological and morphological changes in captive animals” ([17], p. 372). In another study, McPhee [15] also noted changes in behavioral ecology when big cats were given calf carcasses, which caused a decrease in stereotypical behavior off exhibit and an increase in hiding on exhibit. Although the morphological effects of an unnatural dietary consistency have not been fully explored, dietary supplementation is an important component of psychological enrichment [17].

### Previous Studies on the Morphological Effects of Captivity

Although zoos, aquariums, and sanctuaries attempt to provide foods that mimic the nutrition felids would acquire in the wild [18], nutrition is not the only criteria a captive diet must meet in order to fully satisfy a wild animal’s physiological requirements: the consistency and texture–or, the mechanical properties–are also important components of an animal’s diet, as they affect both the dental health and cranial morphology of the animal [19], [20]. A diet that more closely mimics that which would be found in nature could prevent negative changes caused by the physiological under-stimulation pervasive in captive settings [21]. For example, despite receiving adequate nutrition, captive carnivores often experience more dental problems than their wild counterparts [19] due to the lack of abrasive action that accompanies chewing bones [22]. Such abrasive action, which cannot occur with soft, commercial foods, has been thought to serve as a cleaning mechanism capable of preventing plaque buildup [18]. This effect has been shown across species. A study by Gawor and colleagues [20] on 9,074 cats and 29,702 dogs found that dry food positively affects oral health, and that animals that were fed dry food as opposed to wet or a combination of dry and wet had significantly less periodontal pathologies, deposits, and lymphadenopathy. This has been shown in exotic species as well. For instance, in one study [22], captive Amur tigers (*P. tigris altaica*) have exhibited oral health problems attributable to a diet lacking durophagous mechanical properties. In another study [21], palatal erosion in cheetahs has also been associated with captivity status.

Many studies have reported on the impact of captivity on specific pieces of anatomy in captive animals. For instance, cranial thickening has been documented in captive subadult baboons (*Papio* sp.; [23]) and lion cubs [24]. Bone disease attributed to their diet has also been documented in South American primates [25]. One study [26] found lion cub cranial dimensions to be greater in captive than wild individuals, and chinchillas (*Chinchilla laniger*) have also shown to have increased cranial dimensions and variations in skull shape in captivity [27]. Other studies, however, have shown decreased skull sizes of some captive mammals including in Indian rhinos (*Rhinoceros unicornis*; [28]) and some equids (*Equus* spp.; [29]). Increased overall body size relative to maturity rate in captivity has been observed in chimpanzees (*Pan troglodytes*; [30]), yellow baboons (*P. cynocephalus*; [31]), silver foxes (*Vulpes vulpes*; [32]), and callitrichids including golden-headed tamarins (*Leontopithecus chrysomelas*; [19]). Captive Dall’s sheep (*Ovis dalli*) and a male alpine ibex (*Capra ibex*) have both increased body sizes and horns [19], [33]. Studies on pheasant chicks (*Phasianus colchicus*; [34]), old field mice (*Peromyscus polionotus subgriseus*; [35]), squirrel monkeys (*Salmiri sciureus*; [36]), and hyraxes (*Procavia capensis*; [37]) have shown, to varying degrees, nutrition’s impact on bone morphology in general and cranial shape more specifically. Similar shape changes have been observed in reptiles too. For example, broader, flatter skulls were observed in captive American alligators (*Alligator mississippiensis*; [38]), and another study found that durophagous lizards have significantly different head shapes and sizes when compared to lizards that prey on softer organisms [39].

Indirect “domestication” (physical changes resulting from multiple generations in captivity, possibly rendering animals less fit for future reintroduction into the wild) of traditionally wild animals impacts their morphology and could cause pathologies, among other changes [19]. Multiple studies show differences in animals’ skull shapes and sizes as related to the mechanical properties of their diets [19], [39], [40] and captivity status [19], [41]–[43]. In the most thorough survey of these, O’Regan and Kitchener [19] discuss some of the differences between captive and wild morphology. For example, in some felids they note dental pathology, differences in skull shape, and cranial thickening, and other mammals showed reduced sexual dimorphism, which they note may prevent successful integration of reintroduced animals with wild populations [16], [19]. Following inquiries surrounding brain size, Yamaguchi and colleagues [42] conducted a study on captive lion and tiger encephalization, showing reduced brain size in captive felines compared to their wild counterparts. Similarly, Zuccarelli [44] found differences in the morphology of the zygomatic arches of wild and captive lions, with captives displaying increased zygomatic breadths, and most recently, Saragusty and colleagues [45] found differences in the height of the foramen magnum in wild and captive lions (though not tigers). The findings of Hartstone-Rose and colleagues [40], [46]–[48] support the hypothesis that the diet of carnivores will ultimately affect their muscular, as well as osteological and dental masticatory architecture. Through analysis of masticatory muscles and dental architecture, these studies have found links between the mechanical properties of foods that the species consume and bite force and gape abilities as well as tooth shape.

While several studies have examined specific aspects of captive pantherine cranial morphology, this research has generally focused on relatively few variables [45], [49] with a specific interest, most recently on a peculiar foramen magnum stenosis found in captive lions [3], [24], [42], [45], [50]–[52] (but, interestingly, not tigers; [45]) that almost certainly has nothing to do with the mechanical properties of their diet.

In the most comprehensive examination of the differences between captive and wild felid crania, O’Regan and Turner [43] hypothesized that mechanical diet is the cause of morphological differences between wild and captive carnivores. Using ten cranial and seven mandibular measurements (replicated and expanded in the present study) from ninety-seven leopards (*P. pardus*) and lions, they found significantly wider muzzle breadths in captive leopards. Additionally, the zygomatic arches and muzzles in captive lions were found to be larger than in their wild counterparts. Upper and lower carnassial measurements followed the opposite trend in male lions; they are shorter in captive individuals. Because both the muzzle and zygomatic arch are areas important for mastication and masticatory muscle origins respectively, O’Regan and Turner hypothesize that the lack of tearing and biting required of captive felids is a possible cause for these morphological differences.

Our research expands on these previous studies by 1) being the first to examine trends observed in previous studies of lions to a broad sample of tigers – a very similar felid equally represented in captivity, 2) replicating the linear measurements of the most thorough study to date [43], 3) adding key ratios that better describe the qualitative differences previously observed [43], [45], and 4) being the first to use three-dimensional geometric morphometric analyses to examine the shape differences between captive and wild animal populations.

### Hypotheses

While some studies (e.g., [18]) have shown that the consistency and texture of a diet lacking the mechanical properties of a wild diet can have adverse long-term effects on captive carnivores, no study has evaluated these effects using 3D geometric morphometric techniques. Digitizing our sample will allow for a more inclusive study, statistically accounting for minor differences across specimens that are missed by basic observation and giving a more comprehensive view of gross shape differences unattainable through simple comparison of linear data. Using Principal Component Analysis (PCA), we can explore the morphological similarities and differences across a population of mixed captivity statuses and make inferences about the impact of captivity.

Because of the morphological differences between lions and tigers [8], [53], we hypothesize that (H1) one of the main sources of variation will separate the two species. Furthermore, we hypothesize that (H2) another major source of variation will separate the sexes of these two sexually dimorphic species [8], [54], [55]. A PCA of linear variables of tiger skulls by Mazak [54] found that sex accounted for over seventy-seven percent of intraspecific variation. According to Mazak [54], a large portion of the factors influencing the differences across sexes is related to morphology that is strongly correlated with predatory function, such as the shape of the rostra and zygomatic arches. Naples and Rothschild [55] also found substantial sex differences in the porosity of the lion skulls. This difference was attributed to the males’ need to mature quickly and engage in mate competition with other males. This sexually dimorphic porosity is not found in tigers or any other felid [55].

While we expect much of the variation in cranial shape within our sample to describe specific and sex differences, we also hypothesize that (H3) differences in cranial shape will separate captive and wild individuals. Thus, we will evaluate the effect of captivity relative to the morphological contribution of species and sex on the overall cranial geometry.

## Methods

We collected data on all complete adult lion and tiger specimens (Table 1) from the American Museum of Natural History (AMNH; New York), and the Smithsonian Institution’s National Museum of Natural History (USNM; Washington DC) as well as a few specimens from captive animals that were donated to the PI for research purposes by Carolina Tiger Rescue (Pittsboro NC) – these specimens are freely available for study upon request. The sample population of specimens was sorted according to captivity status, species, and sex.

**Table 1:** Sample Carnivore Population (N = 89).

	Captive	Wild	Total
*Panthera leo*			
Males	10	10	20
Females	9	14	23
*Panthera tigris*			
Males	16	9	25
Females	11	10	21
**Total**			**89**

As with previous studies [43], [45], all individuals listed as having come from specific zoos were categorized as “captive”. We also included in this group specimens from other captive collections (e.g., a circus and a rescue center). Zoo specimens listed as having been caught in the wild were excluded in our study. Only specimens with known wild geographic origin were categorized as “wild”.

The wild specimens were collected throughout the natural range of each species and the captive animals were accessioned from The Barnum and Bailey Circus, the Carolina Tiger Rescue, National Zoological Park (Smithsonian), Toledo Zoological Society, and several historical zoos that now fall under the Wildlife Conservation Society (NY): the Bronx Zoo, Central Park Zoo, New York Park Commission, New York Zoo, New York Zoological Gardens, New York Zoological Society, and Prospect Park Zoo).

**Figure 1:**
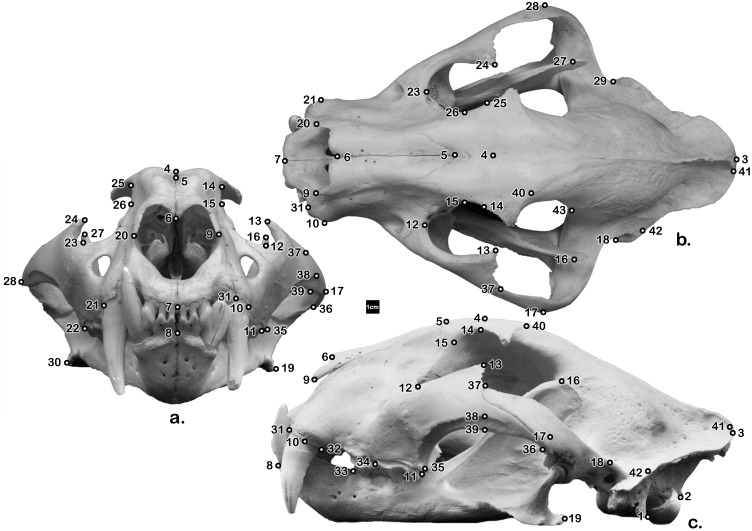
Forty-three landmarks; anterior (a), superior (b), and lateral (c) views. Note that some of the landmarks (e.g., 43) are especially hard to visualize – please see their description (Table 2). Captive ♀ tiger, SCMed Comparative Anatomy Lab Research Collection, University of South Carolina School of Medicine.

Forty-three landmarks (Table 2, Fig. 1) on each specimen were digitally recorded directly onto a spreadsheet with a MicroScribe 3D Digitizer (Solution Technologies, Inc.) recording three-dimensional (x, y, and z) coordinates.

**Table 2:** The forty-three landmark points measured with the MicroScribe 3D Digitizer (Fig. 1).

	Landmark/AnatomicalPoint	Description
1	Foramen MagnumVentral	Median point on the ventrallip of the foramen magnum
2	Foramen MagnumSuperior	Median point on the superiorlip of the foramen magnum
3	Inion	Caudal-most point of the occipital protuberance
4	Vertex	Dorsal-most point along themidline of the neurocranium
5	Nasion	Convergence of the L and R frontal and nasal bones
6	Rhinion	Anterior-most convergence of the nasal bones
7	Alveolare	Anterior-most point on the premaxillary suture,between the alveoli of the left and right central maxillary incisors
8	Infradentale	Anterior-most point on the mandibular symphysis between the alveoli of the left and right central mandibular incisors
9	Antero-lateral nasalcorner L	Antero-lateral-most point on the L nasal bone
10	Buccal edge of maxillaat Canine L	Lateral-most point of L maxillary canine,where it enters the alveolus
11	Distal P4 L	Distal-most point of the metaconeon the fourth L maxillary premolar (P^4^)
12	Orbitale L	Ventral-most point along the bony rim of the L orbit
13	Lateral orbit L	Dorsal-most point on the L zygomatic (jugal) bone L
14	Superior orbit L	Dorsal-most point along the bony rim of the L orbit
15	Medial orbit L	Medial-most point along the bony rim of the L orbit
16	Coronion(Coronoid tip) L	Dorsal-most point of theL coronoid process of the mandible
17	Zygion L	Lateral-most point of the skull on theL zygomatic arch
18	Porion L	Dorsal-most point of the bony rim of theL external auditory meatus
19	Tip of mandibularangle L	Caudal-most point of the L mandibular angle
20	Antero-lateralnasal corner R	Same as Point 9 on the R side
21	Buccal edge ofmaxilla at Canine R	Same as Point 10 on the R side
22	Distal P4 R	Same as Point 11 on the R side
23	Orbitale R	Same as Point 12 on the R side
24	Lateral orbit R	Same as Point 13 on the R side
25	Superior orbit R	Same as Point 14 on the R side
26	Medial orbit R	Same as Point 15 on the R side
27	Coronion(Coronoid tip) R	Same as Point 16 on the R side
28	Zygion R	Same as Point 17 on the R side
29	Porion R	Same as Point 18 on the R side
30	Tip of mandibularangle R	Same as Point 19 on the R side
31	Anterior edge of Canineat premax/max suture L	Anterior-most point on the L maxillary canine, at thepremaxillary/maxillary suture where the tooth enters the alveolus
32	Posterior edge ofCanine L	Posterior-most point of theL maxillary canine, where the tooth enters the alveolus
33	Anterior edge oflower p3 L	Anterior-most point of mandibularL third premolar (P_3_)
34	Anterior edge of P4 L	Anterior-most point of L maxillarycarnassial; fourth maxillary premolar (P^4^)
35	Anterior edge ofmasseter origin L	Ventral-most point along the anteriorextension of the L masseter origin scar
36	Posterior edge ofmasseter origin L	Ventral-most point along the posteriorextension of the L masseter origin scar
37	Superior edge ofzygomatic arch at suture L	Dorsal-most point of theL zygomatico-temporal suture
38	Superior edge of masseterorigin at thickest L	Dorsal-most point of the L masseter origin scarwhere the scar is at its thickest vertical measurement
39	Ventral edge of masseterorigin at thickest L	Ventral-most point of the L masseter origin scarwhere the scar is at its thickest vertical measurement
40	Anterio-superior cornerof temporalis origin L	Point on the dorsal surface of the L frontal bone, justbehind the superior process of the orbit along the ridge of the temporal line
41	Posterio-superior cornerof temporalis origin L	Point on the most posterio-superior corner of the Lparietal along the ridge of the temporal line
42	Posterio-inferior cornerof temporalis origin L	Ventral-most point on the L temporalis origin scardorsal to the mastoid process
43	Anterior-inferior cornerof temporalis origin L	Point located on a small process just lateralto the L optic foramen

The three-dimensional geometric morphometric analysis programs, Morphologika, (version 2.5) and MorphoJ (version 1.05f) were used for statistical analysis. We calculated linear distances between several of the points using the Pythagorean Theorem to compare our findings with previous studies that did not use three-dimensional analyses (e.g., [43], [56]), and we expanded upon these previously discussed measurements based on trends that emerged in our three-dimensional analyses. We also added two ratios: Alveo-orbital:Inioorbital (a proxy for the rostrum length relative to the neurocranium length) and Bicoronal:Biangular (a proxy for the angle of the ascending ramus) (Table 3). Using JMP (version 10.0.2) we conducted PCA and ran one-way analyses on all linear metrics with respect to captivity status, sex, and species. All results were considered statistically significant for alpha <0.05.

**Table 3:** Measurements Taken From Landmark Coordinates.

	Measurement	Landmarks^a^
I	Alveo-orbital Length	7 to 12
II	Basal L^b^	1 to 8
III	Biangular (BA)	19 to 30
IV	Bicoronal (BC)	16 to 27
V	Canine To Condyle L^b^	1 to 10
VI	Coronoid H^b^	16 to 19
VII	Iniorbital Length	3 to 12
VIII	Interobital Distance^b^	15 to 26
IX	L Of P4^b^	11 to 34
X	Mandible L (Infradentale-Corion)	8 to 16
XI	Mandible L (Infradentale-Angular)	8 to 19
XII	Muzzle Breadth^b^	11 to 22
XIII	PM Row L^b^	11 to 33
XIV	Rostral Breadth^b^	10 to 21
XV	Rostral Length	5 to 6
XVI	Skull Length	3 to 7
XVII	Zygomatic Breadth^b^	17 to 28
XVIII	Alveoorbital:Inioorbital Ratio	I/VII
XIX	Bicoronal:Biangular Ratio	IV/III

**Figure 2:**
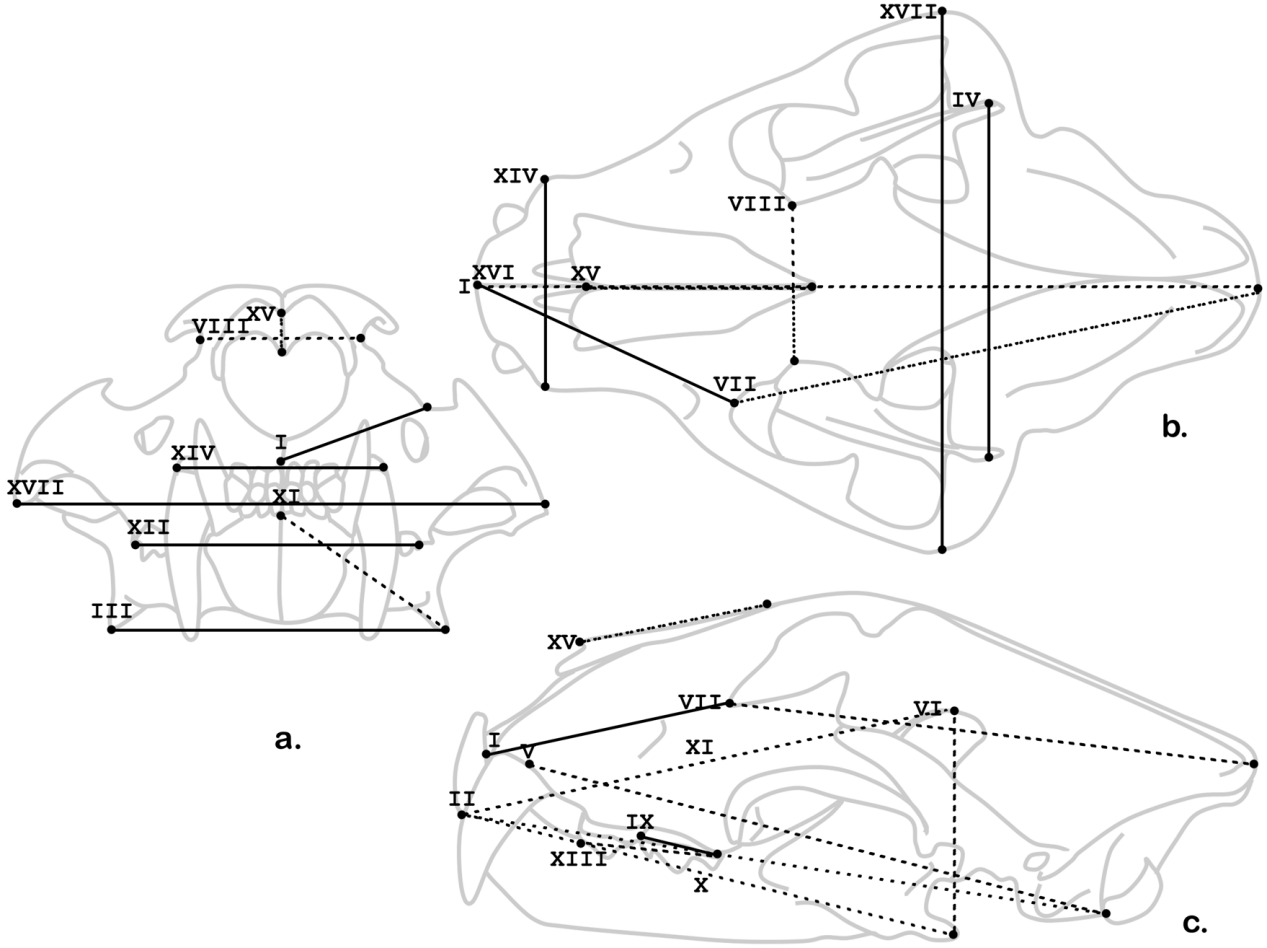
Linear variables (see table 3 for description) that significantly (solid) divide the sample by captivity status. (The other variables that do not distinguish captive from wild specimens are included as dashed lines).

See Fig. 2 for graphic representation of these variables.

a See Table 2 for description of landmarks.

b Measurements described in [56].

Principal Component Analysis (PCA) is a statistical procedure through which a set of possibly correlated variables is transformed into a new set of orthogonal variables (termed “Principal Components”; PCs) in which the first Principal Component (PC1) describes the most variance in the sample and successive PCs describe sequentially less. Essentially, the analysis is a statistically valid way to evaluate which of a large set of variables is driving the variation in a sample and yields information about how much each variable contributes to that variation. PCA is often employed in morphological studies to deduce which measurements drive the variation in a sample. For instance, in our study, we measured 17 distances between points on the specimens (Table 3) that we thought would describe the variation in our sample. PCA tells us which of these vary most significantly – i.e., which are driving the variation in the sample. PCA can also be employed on more abstract data sets; for instance, in our study PCA is used to analyze the raw three-dimensional data clouds resulting in the “lollipop” diagrams that show which data points most influence the variation in terms of both magnitude and direction (see results for an explanation of these diagrams).

In Morphologika we used Procrustes fit to digitally superimpose (rotate and translate) the specimens so that they could be compared to each other in aligned space “minimizing the sum of the squared distances between corresponding landmarks” ([57], p. 24). Procrustes allows for analysis of the shape changes of each specimen relative to the sample, taking size into account. Although scaling the sample (a simple option in Morphologika’s Procrustes fit) would restrict the three-dimensional analyses to shape variation, we regard size to be an important factor. Sex certainly is expected to influence skull size in these dimorphic taxa, but so too may captivity status if factors like starvation (which could restrict growth) or dietary abundance (which might increase growth) were to affect the population. Thus, we never scale our variables prior to analysis, but rather consider the size effect (almost always the first source of variation) along with the subsequent shape effects.

## Results

### Analyses of Linear Variables

Males and Females are statistically distinct across each of the linear variables (I-XVII), as we hypothesized (H2); males are substantially larger than females in all linear measures (Table 4). However, the sexes do not differ by either of the shape ratios. Those ratios clearly separate lions from tigers as lions have significantly longer rostra and narrower biangular widths – thus supporting H1 as well. Although the upper carnassial (P4) and premolar-molar rows in lions are only slightly longer than those of tigers (35.57 mm vs. 33.79 mm and 68.25 vs. 63.12 respectively), these differences were also highly significant. All of the statistically significant differences including these tooth lengths along with basal skull length (II), two different metrics related to jaw length (V, and X), and the aforementioned mentioned rostral lengths (I and XIV), all relate, essentially, to the lion’s overall longer muzzle while the tiger has a significantly wider rostrum (XV). (Table 4).

**Table 4:** Output from one way analysis of variance with of linear variables and PCs by group.

	Variable	Sex	Species	Captivity
I	Alveo-orbital L	<0.0001*	<0.0001*	0.0184*
II	Basal L	<0.0001*	0.0408*	0.1434
III	Biangular (BA)	<0.0001*	0.0002*	0.0007*
IV	Bicoronoid (BC)	<0.0001*	0.3491	0.0006*
V	Canine to Condyle L	<0.0001*	0.0196*	0.1358
VI	Coronoid H	<0.0001*	0.4244	0.7356
VII	Inio-orbital L	<0.0001*	0.399	0.7339
VIII	Interorbital Distance	<0.0001*	0.774	0.3662
IX	L of P4	<0.0001*	0.0012*	0.0115*
X	Mandible L (Infradentale-Coronion)	<0.0001*	0.0098*	0.0736
XI	Mandible L (Infradentale-Angular)	<0.0001*	0.1518	0.0586
XII	Muzzle Breadth	<0.0001*	0.1528	0.0181*
XIII	PM Row L	<0.0001*	<0.0001*	0.3179
XIV	Rhinion to Nasion	<0.0001*	0.0059*	0.2172
XV	Rostral Breadth	<0.0001*	0.0226*	0.0118*
XVI	Skull L	<0.0001*	0.2343	0.2272
XVII	Zygomatic Breadth	<0.0001*	0.5663	0.0044*
XVIII	Alveo-orbital:Inioorbital Ratio	0.1194	<0.0001*	0.0066*
XIX	Bicoronal:Biangular Ratio	0.4122	<0.0001*	0.3141
	PC1 (Linear Measurements)	<0.0001*	0.2091	0.0996
	PC2 (Linear Measurements)	0.3417	<0.0001*	0.0013*
	PC3 (Linear Measurements)	0.7007	0.0002*	<0.0001*
	PC4 (Linear Measurements)	0.653	0.0596	0.1478

*denotes statistically significant results.

Hypothesis 3 (H3) is also supported – there are statistically significant differences between captive and wild pantherines. As has been found by many authors previously [1]–[3], [19], [43], [45], [55], [56] zygomatic arch width statistically differentiates captive and wild lions and tigers with captive specimens having significantly wider skulls. This difference is more than a centimeter on average and the 95% confidence intervals do not overlap – thus the widely acknowledged qualitative observation has clear quantitative validity. However, it is not the most statistically significant differentiator of captive and wild animals; both of the measures of mandibular width – bicoronoid and biangular widths – are also more than a centimeter wider in captive animals with even less overlap between the groups (Table 4, Fig. 2). Thus, not only do the zygomatics flare, as has been previously noted, but so too do the mandibles. Captive animals also have significantly longer alveo-orbital distances, but shorter carnassials, and wider rostra and muzzles. (Table 4, Fig. 2).

**Figure 3:**
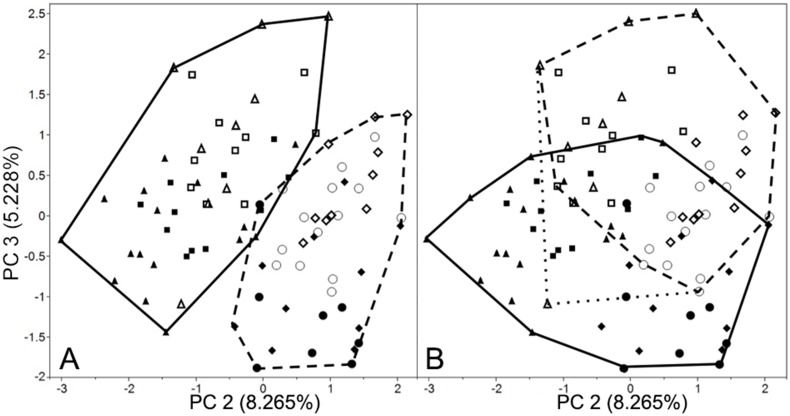
PCA output with second principal component (x-axis) against third (y-axis) from analysis of linear variables. Minimum convex lines describe: A. tigers (solid) and lions (dashed); B. captives (solid) and wilds (dashed) with a single wild outlier AMNH 85396 (dotted). Markers represent female tigers (squares), male tigers (triangles), female lions (circles), and male lions (diamonds). Wild specimens are represented by open markers and captive specimens by closed markers. See Table 5 for linear PCA scores.

When all of the linear variables are entered into a principal component analysis, the first principal component (accounting for 72.7% of the variation), as expected for unscaled values, is strongly driven by overall size (as indicated by the positive sign of all of the eigenvectors; Table 5) and statistically separates the sample only by sex (and not species or captivity status; Table 4). Although the second and third principal components (accounting for 8.3% and 5.2% of the variation respectively; Table 5) both significantly divide the sample according to species and captivity status (and not sex). PC 2 divides the sample more clearly by species (Fig. 3A) while PC 3 divides the population more clearly by captivity status – especially when one outlier is removed (Fig. 3B). (The removal of this outlier does not affect the statistical significance of the findings.) The subsequent principal components (accounting for slightly more than 10% of the variation) do not differentiate any of the groups with any clear pattern.

**Table 5:** Principal Component eigenvalues and eigenvectors of linear variables.

	PC 1	PC 2	PC 3	PC 4
Eigenvalue	12.3618	1.4051	0.8888	0.618
Percent	72.717	8.265	5.228	3.635
Cumulative Percent	72.717	80.982	86.21	89.846
Eigenvectors				
Alveo-orbital L	0.23795	0.28059	–0.26681	–0.14133
Basal L	0.27649	0.08258	–0.05699	–0.14588
Biangular (BA)	0.19537	–0.51181	–0.11387	0.23412
Bicoronoid (BC)	0.22371	–0.17524	–0.44474	0.08058
Canine to Condyle L	0.27144	0.07291	–0.09792	–0.25517
Coronoid H	0.23737	–0.06289	0.09497	–0.47065
Inio-orbital L	0.26442	–0.14824	0.18219	–0.17860
Interorbital Distance	0.23403	–0.04994	0.16070	0.23918
L of P4	0.17794	0.46578	0.27676	0.33309
Mandible L (Infradentale-Corion)	0.26836	0.12171	–0.12392	–0.09087
Mandible L (Infradentale-Angular)	0.27920	0.01497	–0.05844	–0.71980
Muzzle Breadth	0.24670	0.03044	–0.13137	0.39654
PM Row L	0.20935	0.49200	0.01817	0.14387
Rhinion to Nasion	0.18157	–0.17004	0.70853	–0.10114
Rostral Breadth	0.24260	–0.17621	0.12757	0.41110
Skull L	0.27869	–0.00295	0.02671	–0.19454
Zygomatic Breadth	0.26114	–0.23042	–0.07226	0.04803

The second principal component of the linear variable PCA is driven, predominantly, by an inverse relationship of the rostral lengths (most substantially the carnassial length and premolar-molar row length) to the skull width variables (most substantially the biangular and zygomatic widths) – as expected for the long muzzles typical of lions relative to the wide skulls more characteristic of tigers. PC 3 is driven most substantially by an inverse relationship between the rhinion to nasion length and the bicoronoid width. Thus, although the minimum convex units (Fig. 3B) visually separate the population according to captivity status, the eigenvectors that drive this axis are somewhat different than the variables that most substantially sort according to captivity status on their own. The fourth principal component, which does not statistically differentiate the sample by any group (Table 4), is driven primarily by an inverse relationship in the rostral and muzzle breadths relative to some of the mandibular metrics. (Table 5).

### Analyses of Three-Dimensional Variables

**Figure 4:**
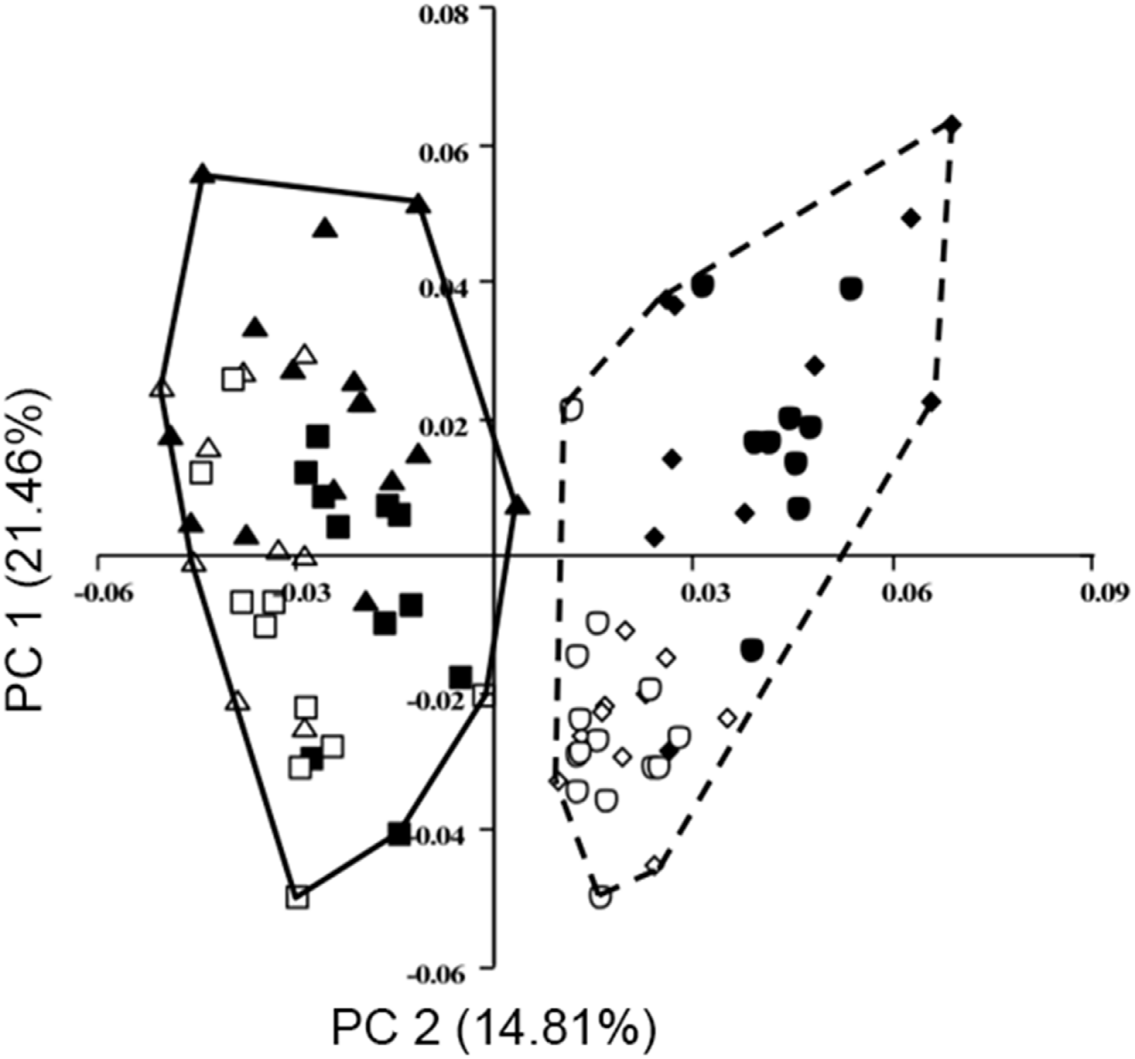
PCA output with first principal component (x-axis) against second (y-axis) from analysis of three-dimensional coordinates. Minimum convex lines describe tigers (solid) and lions (dashed). Key same as in Fig. 3.

**Figure 5:**
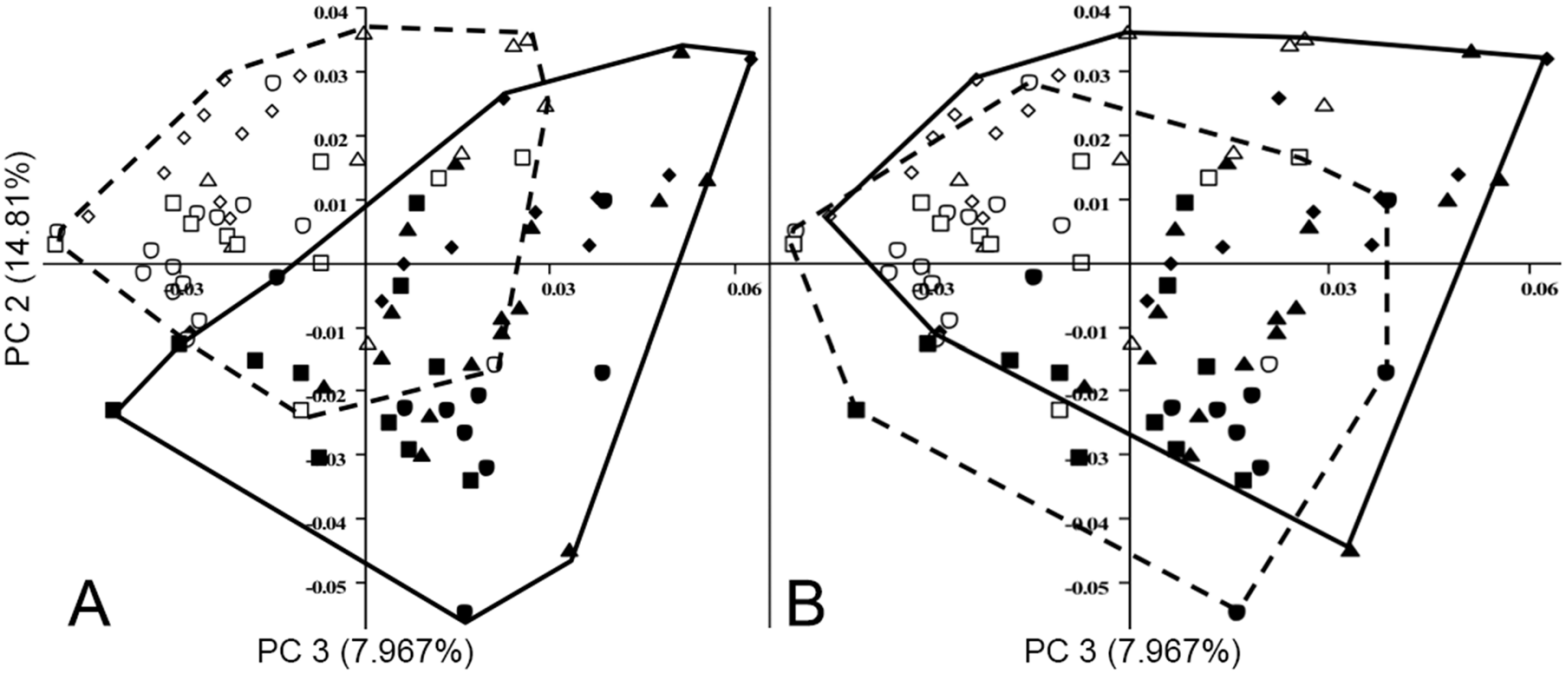
PCA output with second principal component against third, from analysis of three-dimensional coordinates. Minimum convex lines describe: A. captive (solid) and wild (dashed); B. male (solid) and females (dashed). Key same as in Fig. 3.

The results of the three-dimensional analyses (unique to this study as an examination of the effects of captivity) tell a more surprising story: although the first principal component (accounting for 21.46% of the variation; Table 6) divides the population perfectly according to species (Fig. 4), the second principal component (accounting for 14.81% of the variation; Table 6) divides the sample more according to captivity status (Fig. 5A) while the third principal component divides the sample more according to sex (Fig. 5B). Thus, in terms of three-dimensional shape, lion and tiger skulls can be perfectly separated (PC1) and contrary to our expectations the second greatest source of variation separates the sample according to captivity status and not sex. Although there is significantly more overlap in the groups in the subsequent principal components, almost twice as much variation is driven by PC2 than PC3– and thus captivity status affects skull shape much more than sex does in these highly sexually dimorphic species.

**Table 6:** Three-Dimensional PCA Eigenvalues and Percent Variances.

	Eigenvalue	Total Variance (%)	Cumulative Variance (%)
PC 1	9.954E-04	21.46	21.46
PC 2	6.872E-04	14.81	36.27
PC 3	3.696E-04	7.967	44.24
PC 4	2.844E-04	6.131	50.37
PC 5	2.120E-04	4.570	54.94

**Figure 6:**
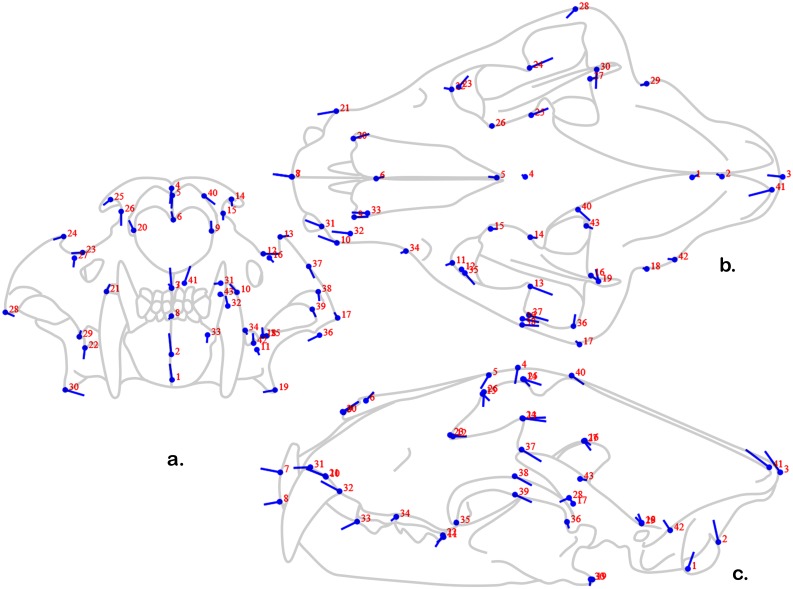
“Lollipop” diagram of PC 1 shape changes in three-dimensional data. Anterior (a), superior (b), and lateral (c) views. The dots (“candy”) represent the shape at the origin and the lines (“sticks”) indicate the shape change in the positive direction along the axis.

The first principal component is driven most substantially by the anterior-most points relative to the position of the points that lie most close to the midline of the skull in the lateral view – i.e., the position of the zygomatics and the post-orbital processes (Fig. 6). Given that this axis divides the population by species, it is not surprising that the variables that emerge describe the relatively longer muzzle of lions relative to tigers. What is somewhat contrary to what we would have predicted both the anterior-most *and* posterior-most points show an anterior shift from the tiger morphospace (represented in Fig. 6– by the dot) to the lion morphospace (represented by the end of the line emerging from the dot). Thus the longer rostra found in lions is driven not by an elongation of the anterior portion of the skull, but by the relatively posterior position of the zygomatics and orbits. In other words, according to this analysis, tigers do not have relatively shorter snouts, but relatively rostral eyes and cheeks.

**Figure 7:**
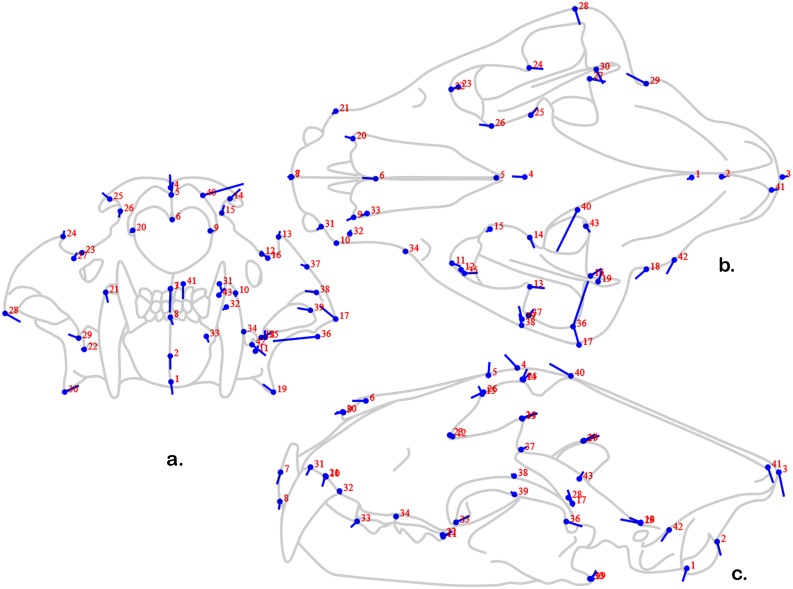
“Lollipop” diagram of PC 2 shape changes in three-dimensional data. See Fig. 6 for explanation of “Lollipop” diagram.

The second principal component is driven by a ventral shift in the anterior- and posterior-most points and a dorsal shift in the dorsal-most points (Fig. 7). The lateral-most points are also greatly affected – a finding in accordance with the conclusion that this axis has a strong relationship with captivity status. Some of the other points that shift substantially along this axis relate to the temporalis origin (i.e., points 40–43) especially the location of the antro-superior corner – the most dramatically shifted point other than those of the zygomatic arch. If we take this axis to be most related to captivity status, captive animals have wider skulls that are less domed than their wild counterparts with dramatically differently shaped temporalis origin – suggesting the importance of this masticatory muscle in the overall shape change in this axis.

**Figure 8:**
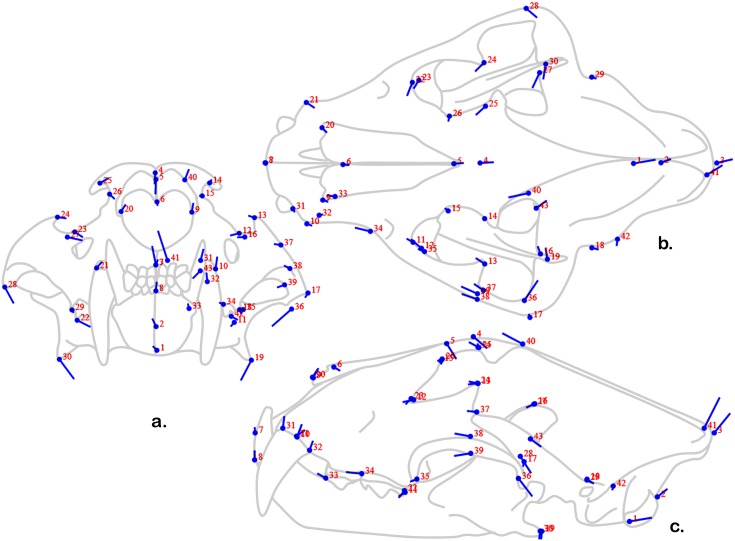
“Lollipop” diagram of PC 3 shape changes in three-dimensional data. See Fig. 6 for explanation of “Lollipop” diagram.

The third principal component is driven by a ventral shift in the mandibular angle and posterior origin of the masseter, and anterior shift in the mid-zygomatic points, as well as slight to moderate postero-dorsal shifts in the canine and occipital points (Fig. 8). Also, the temporalis origin points are drastically different. Although the population overlaps greatly along this axis (Fig. 6B), if this axis is regarded as relating most substantially to sex, then females (represented by the dots) have slightly lower occiputs and canine alveoli, more posterior orbits and zygoma, and substantially less dorsally flared mandibular angles and posterior zygomatic roots. The difference in temporalis origin also implies that females have relatively smaller masticatory muscles.

**Figure 9:**
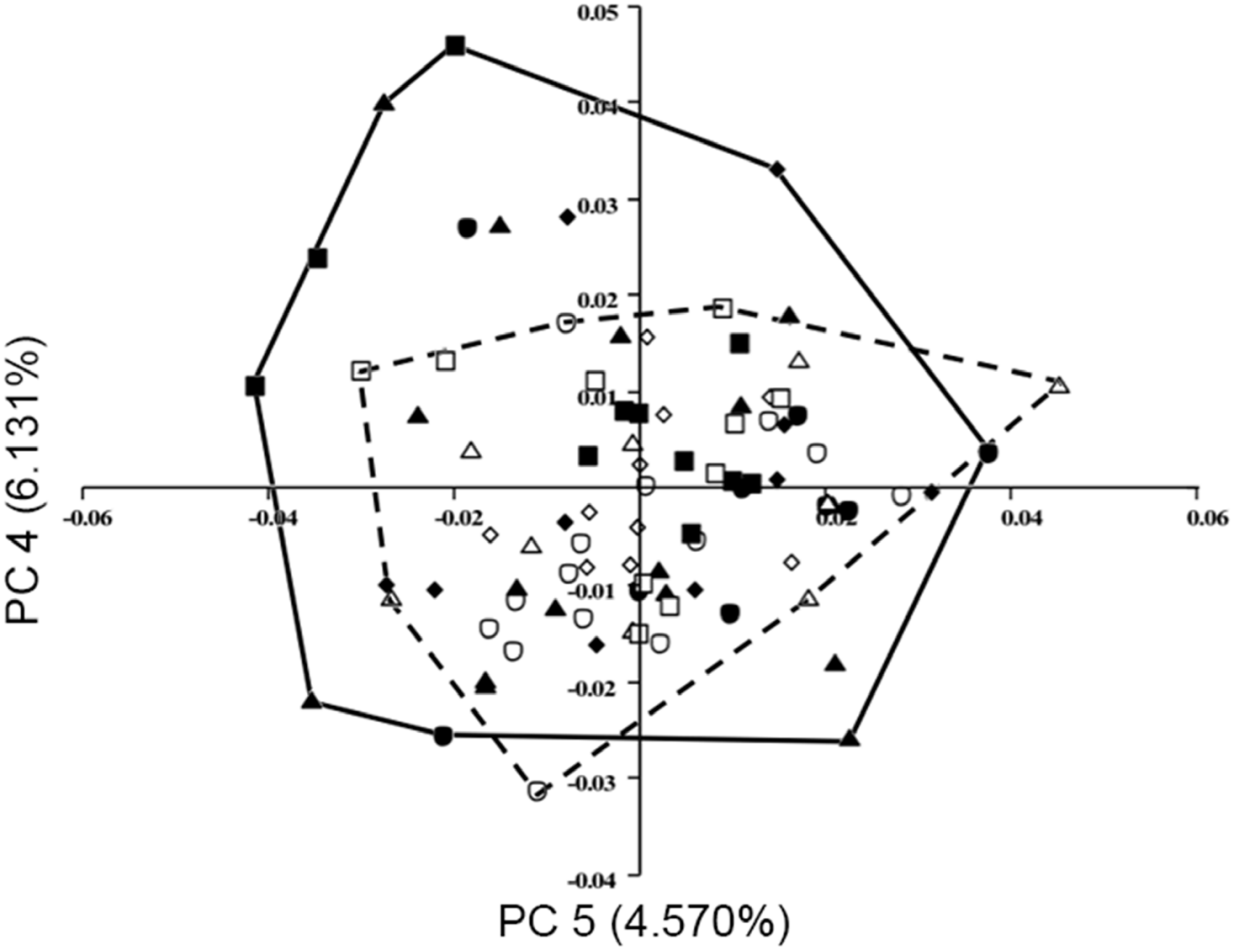
PCA output with fourth principal component against fifth, from analysis of three-dimensional coordinates. Minimum convex lines describe captive (solid) and wild (dashed). Key same as in Fig. 3.

Although no strong morphological pattern was evident in analysis of the fourth and fifth principal components that resulted from the three-dimensional data (lollipop diagrams not shown), it is interesting to note that along these axes, the captive specimens occupy much more morphospace than the wild specimens do (Fig. 9). In fact, only two wild specimens fall outside of the captive morphospace, while more than a dozen of the captive specimens fall outside of the wild morphospace. Thus, in these axes (and almost every other examined), the captive population exhibits broader morphological diversity than does the wild group.

## Discussion

A lion or tiger’s captivity status influences the three-dimensional shape of the skull almost twice as much as its sex does. While we expected to see strong specific and sexual signals from our data, the extent to which captivity status affected morphological variation, particularly in the analysis of the three-dimensional shape variation, was more extreme than we anticipated. Qualitatively, captive big cat skulls look different, and quantitatively, we knew that some key variables bore this out. However, the fact that captivity status related most closely to the second most important source of three-dimensional variation (PC 2), a variable accounting for nearly twice the amount of variation as the axis that most closely tracked with sexual dimorphism (PC 3) was unexpected.

The analyses of both the linear and three-dimensional data yielded significant results for dividing the sample by species, sex and captivity status. Lions and tigers, though similar in body size and ecology are certainly morphologically distinct and strongly sexually dimorphic [8]; both distinctions were clearly seen in the variables included in our study. However, in the prior research literature, the morphological distinctiveness of captive animals was less certain; although many morphologists have routinely excluded captive specimens from their samples based on qualitative impressions of distinctiveness, few studies have quantified these differences, and those that have, have found only a few significant variables. For the taxa in our study, the previously examined variables that relate to captivity status are most notably foramen magnum constriction in lions (but not tigers; [3], [45]) and wider zygomatic arches and cranial thickness (again described most commonly for lions with less information about other felids [1], [2], [19]). Furthermore, the only study to have examined the effects of captivity on overall three-dimensional skull shape focused on a reptile [38] – the necessity to confirm these effects in mammals was still outstanding.

Whether these differences are due to mechanical properties of diet is a persistent question. The fact that we observe important variation in the shape of the temporalis origin (Fig. 7) seems to support the hypothesis that at least some of the differences between captive and wild animals are due to masticatory factors. So too could the extensively noted differences in zygomatic arch breadth; not only is this the origin on the masseter muscles – the second largest mandibular adductor group, but the temporalis muscles run deep to this anatomy. More greatly flared zygomatic arches might indicate that captive lions and tigers have larger temporalis muscles (i.e., necessitating more space between the zygomatic arches and the neurocranium), though the differences in the shape of the temporalis origin (Fig. 7) seem to contradict this. Likewise, there seems to be a mixed signal in the morphology of the masseter origins: the wider zygomatic arches and the more ventrally flared mandibular angles seem to indicate that the masseter muscles could be more massive in captive animals (i.e., as the difference in these two regions – the origin and insertion – increase then the length and probably the thickness also do), but with increased zygomatic widths, the masseters become more oblique to the sagittal plane and therefore more of the pull of the muscle is out of the plane of the mandibular adduction.

Are the masticatory muscles of captive lions and tigers bigger than those of wild animals? Is the fiber architecture different – e.g., might captive animals have larger, though less pinnate muscles [25]? Because of sampling difficulties (i.e., the prospect of getting a statistical sample of the soft tissue of wild specimens of these taxa) these are difficult questions to answer. However, an examination of another taxon widely represented in captivity and available from the wild (e.g., in North America, otters, bobcats, wolves) might give us an avenue to evaluate the effects of captivity on soft-tissues.

More importantly, although we hypothesize that the shape differences that we and others have observed are driven at least partly by dietary differences, other differences between captive and wild pantherines (e.g., the differences that correlate to neuroanatomy; [3], [42], [45], [50], [51]) seem unlikely to be related to the mechanical properties of diet. If the mechanical properties of food do substantially impact cranial morphology, then a species with a diet similar in both captivity and in the wild should exhibit less morphological difference. To test this, future work should focus on a species well represented from both captive and wild specimens; for instance, the California sea lion (*Zalophus californianus*) which consumes primarily fish in both captivity and in the wild [58], [59]. If there are observable differences in the cranial morphology of *Z. californianus* related to captivity status, this would indicate that other factors (such as genetics) are driving this morphological variation – for example, the species may be strongly influenced by genetic problems associated with inbreeding.

If a stronger link is supported between the mechanical properties of diet and cranial morphology, then it will be valuable to increase the sample to include carnivores of substantially different diets (e.g., the omnivorous ursids – fed a wide range of diets in captivity) and non-carnivorans (e.g., an examination of the morphological effects of captivity on primate crania). As this research progresses, these studies could influence and help improve standard husbandry practices with the goal of maintaining captive animals in a more naturalistic state.

In short, this study documents the fact that there are significant differences between captive lions and tigers in more linear measurements than have been previously examined and in the three-dimensional shape of their crania. Although the magnitude of these differences is notable – i.e., by some measures captive animals are more different from wild ones than males are from females in these highly dimorphic species – more work needs to be done to determine the reason for these differences. The differences could not be “adaptive” in an evolutionary sense – there has not been enough time for natural selection to have acted on captive populations and more importantly the breeding and survival of captive animals is anything but natural – but perhaps the relaxation of natural pressures has allowed this morphology to drift in this direction. We hypothesize that the drastically different diet between wild and captive carnivores has been a major factor in these morphological differences, but other factors like inbreeding need to be evaluated in further extensions of this research.

## Supporting Information

Data S1 **Complete primary data file.** Please see text for explanation of the principal component values included from Morphological analysis of 3D cranial points.
(XLSX)
